# Clinical and molecular analyses of norovirus-associated sporadic acute gastroenteritis: the emergence of GII.17 over GII.4, Huzhou, China, 2015

**DOI:** 10.1186/s12879-016-2033-x

**Published:** 2016-11-29

**Authors:** Peng Zhang, Liping Chen, Yun Fu, Lei Ji, Xiaofang Wu, Deshun Xu, Jiankang Han

**Affiliations:** 1 Huzhou Center for Disease Control and Prevention, Huzhou, 313000 China; 2 Huzhou Center for Disease Control and Prevention, 999 Changxing Road, Huzhou, Zhejiang 313000 China

**Keywords:** Norovirus, GII.17, Molecular epidemiology, Acute gastroenteritis

## Abstract

**Background:**

Noroviruses (NoVs) are the most common cause of non-bacterial acute gastroenteritis (AGE) in all age groups worldwide. The NoVs circulating in Huzhou over the past 7 years were predominantly GII.4 genotypes. In the winter of 2014–2015, a novel variant of NoV GII.17 emerged and became predominant. We report the epidemiological patterns and genetic characteristics of NoV after the appearance of GII.17 in Huzhou City, Zhejiang, China.

**Methods:**

Between January and December 2015, 746 stool specimens collected from patients with acute gastroenteritis were screened for NoV. Real-time RT-PCR (qPCR) was performed for NoV detection. RT-PCR was used for genomic amplification and sequencing. Genogroups and genotypes were assigned using an online NoV typing tool (http://www.rivm.nl/mpf/norovirus/typingtool). Phylogenetic analyses were conducted using MEGA (ver. 6.06).

**Results:**

In total, 196 (26.3%) specimens were identified as NoV-positive. NoV infection was found in all age groups tested (≤5, 6–15, 16–40, 41–60, and ≥60 years), with the 16–40-year age group having the highest detection rate (117/196, 59.7%). Of the 196 NoV-positive specimens, 191 (97.5%) viruses belonged to GII, and 4 (2.0%) to GI; one sample showed GI and GII co-infection. Overall, 117 (59.7%) viruses were sequenced, and new GII.P17/GII.17 variants were the dominant genotype, accounting for 75.2%, followed by GII.Pe/GII.4 Sydney 2012 strains (11.11%). AGE patients infected with the GII.P17/GII.17 genotypes almost all had abdominal pain and watery stools.

**Conclusions:**

We report the epidemiological patterns and genetic characteristics of the emergence GII.17 over the GII.4 in Huzhou between January and December 2015. After the emergence of GII.17 in October 2014, it steadily replaced the previously circulating GII.4 Sydney 2012 strain, and continued to be dominant in 2015.

**Electronic supplementary material:**

The online version of this article (doi:10.1186/s12879-016-2033-x) contains supplementary material, which is available to authorized users.

## Background

Norovirus (NoV) is the most common cause of non-bacterial acute gastroenteritis (AGE) in all age groups worldwide [1]. Transmission of NoV is mainly via fecally contaminated food or water, by direct contact with patients or vomited virus, and subsequently by contact with contaminated environmental surfaces [2]. Clinical infection with NoV generally has an incubation time of 12 to 48 h, with nausea, vomiting, watery diarrhea, and abdominal pain [3].

NoV has a diameter of ~38 nm and belongs to a category of small non-enveloped icosahedral viruses in the Caliciviridae family. The genome of NoV consists of a ~7.5-kb positive sense, single-stranded RNA with three open reading frames (ORF1-ORF3) [4]. Genetically, NoV has been classified into six genogroups (I–VI) that are further subdivided into over 40 genotypes [5]. Genogroups I, II, and IV have been found to infect humans, whereas genogroup III infects bovines, and genogroup V has been isolated from mice. The sub-genogroup GII.4 virus accounts for most reported cases, and has been identified as the predominant genotype globally; new GII.4 variants emerge every 2–3 years [6, 7].

Over the past 7 years, the GII.4 genotype, including GII.4 variants 2006b, New Orleans 2009, and Sydney 2012, has been predominant in Huzhou [8, 9]. Although various genotypes have been found among genogroups I and II, including GI.P2/GI.2, GI.P3/GI.3, GI.P4/GI.4, GII.P12/GII.3, GII.P7/GII.6, GII.P16/GII.13, and GII.Pg, none has ever replaced the GII.4 genotype.

In the winter of 2014–2015, a novel variant of NoV GII.17 emerged and became predominant in Huzhou, and steadily replaced the previously circulating GII.4 Sydney 2012 strain [10]. At the same time, GII.17 emerged and became predominant in the United States [11], Europe [12], and other places in Asia [13–16].

Did GII.17 appear in Huzhou temporarily, or did it replace GII.4 forever? Here, we report epidemiological patterns and genetic characteristics of NoV after the appearance of GII.17 in Huzhou City, Zhejiang, China.

## Methods

### Specimen collection

This study was part of the regional NoV gastroenteritis surveillance program conducted at the First People’s Hospital in Huzhou, and was approved by the ethics committee of Huzhou Center for Disease Control and Prevention. Informed consent for the stool samples was obtained from the patients or their guardians.

The definition of acute gastroenteritis was diarrhea (≥3 loose stools within a 24-h period), possibly accompanied by vomiting, abdominal pain, fever, and nausea. All stool samples were freshly collected in a sterile container and sent to Huzhou Center for Disease Control and Prevention for immediate storage at -70 °C prior to analysis.

### Viral RNA extraction and norovirus detection

Viral RNA was extracted from 140 μL of supernatant of a 10% (w/v) fecal suspension using a QIAamp Viral RNA Mini Kit (QIAGEN, Hilden, Germany) according to the manufacturer’s protocol. RNA extracts were subjected to the reverse transcription polymerase chain reaction (RT-PCR) or stored at -70 °C until further use. Genogroup-specific primers and probes described previously were used to detect NoVs by real-time RT-PCR (qPCR) [17]. Primer and probe sets JJV1F/JJV1R/ JJV1P and JJV2F/COG2R/RING2-TP were used to screen for GI and GII NoV strains, respectively. RT-qPCR was carried out using a One Step PrimeScript RT-PCR Kit (DRR064; TaKaRa, Dalian, China). Amplification conditions were described previously [8].

### Genomic amplification for genotyping

For genotyping, the primer set JV12Y/JV13I was used to amplify the 3’-end of the RdRp (RNA-dependent RNA polymerase) gene (region A in ORF1) [18]. Primer sets G1SKF/G1SKR and G2SKF/G2SKR were used to amplify the 5’-end of the capsid protein (VP1) gene (region C in ORF2) for GI and GII, respectively [19]. RT-PCR was carried out using a One Step RNA PCR Kit (TaKaRa) with the amplification conditions described previously [8]. After amplification, 5 μL of the PCR products was visualized by agarose gel electrophoresis. The residual PCR products were purified using a QIAquick PCR purification kit (Qiagen, Leusden, The Netherlands), and the purified products were sequenced directly at both ends with amplification primers by TaKaRa Biotechnology (Dalian, China).

### Sequence analysis and phylogenetic analysis

Genotypes were determined using the online NoV Typing Tool (http://www.rivm.nl/mpf/norovirus/typingtool) [20], and the strains were named according to the isolated place, time, and sample number. A phylogenetic tree was generated using the neighbor-joining method and MEGA software (ver. 6.06) [21]. The evolutionary distance was calculated based on the maximum composite likelihood model, and the reliability of each branch was assessed with 1000 bootstrap replicates.

### Nucleotide sequence accession numbers

The GenBank accession numbers for sequences obtained in this study are KU662973-KU663020.

## Results

### Norovirus infections and clinical features

**Fig. 1:**
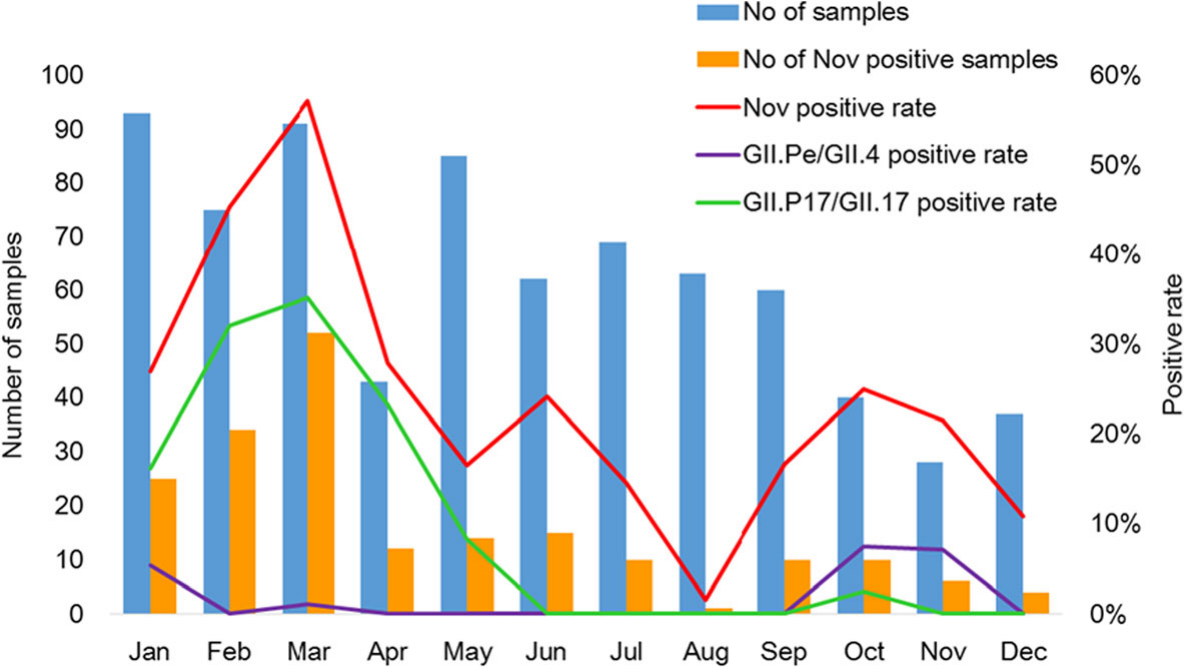
Temporal distribution of norovirus infection from January to December 2015 in Huzhou. Monthly detection rate of norovirus in patients with acute gastroenteritis (AGE) from January to December 2015 in Huzhou, China

Between January 2015 and December 2015, 746 stool specimens collected from patients (including children and adults, outpatients and inpatients) with AGE were screened for NoV. In total, 196 (26.3%) specimens were identified as NoV-positive. Among the 196 positive samples, 191 (97.45%) viruses belonged to GII, 4 (2.04%) to GI, and 1 sample showed GI and GII co-infection. The numbers of AGE patients and the monthly detection rates of NoVs are shown in Fig. 1. The NoV detection rate increased from January, reached a peak in March, and then declined. The highest detection rate was 57% in March, at which point NoV infection accounted for nearly half of all AGE patients. In contrast, the lowest NoV infection rate was in August, when only 1 of 63 samples was positive.

We compared the clinical characteristics of AGE patients infected with NoVs versus those without NoVs (Table 1). NoV infection was found in all age groups tested (≤5, 6–15, 16–40, 41–60, and ≥60 years), with the 16–40-year age group having the highest detection rate (117/196, 59.7%). The female-to-male ratio was 0.94 (95:101) in NoV-positive patients and 94.4% (185/196) of NoV infections were detected in outpatients. The clinical features of the NoV-associated AGE patients were fever (12/196, 6.1%), watery stool (194/196, 99%), abdominal pain (176/196, 89.8%), and vomiting (42/198, 21.4%). There was no statistically significant difference between NoV-positive and -negative AGE patients in terms of sex, age, setting, or clinical features.

**Table 1:** Epidemiological and clinical features of acute gastroenteritis (AGE) patients

	Positive	Negative				
Parameter	*N* = 196	*N* = 550	*χ* ^*2*^	*P*	OR	95% CI
Sex (male:female)	101:95	252:298	1.892	0.169	1.257	0.907–1.743
Setting			2.124	0.145	0.608	0.309–1.194
Inpatient	11 (5.6)	49 (8.9)	–	–	–	–
Outpatient	185 (94.4)	501 (91.1)	–	–	–	–
Age			5.963	0.199	–	–
≤5	10 (5.1)	37 (6.7)	–	–	–	–
6–15	2 (1.0)	12 (2.2)	–	–	–	–
16–40	117 (59.7)	275 (50.0)	–	–	–	–
41–60	46 (23.5)	145 (26.4)	–	–	–	–
>60	21 (10.7)	81 (14.7)	–	–	–	–
Fever (>38 °C)			1.855	0.173	0.638	0.333–1.224
Yes	12 (6.1)	51 (9.3)	–	–	–	–
No	184 (93.9)	499 (90.7)	–	–	–	–
Vomiting			1.371	0.242	1.274	0.849–1.911
Yes	42 (21.4)	97 (17.6)	–	–	–	–
No	154 (78.6)	453 (82.4)	–	–	–	–
Stool type			4.039	0.097	–	–
Watery	194 (99.0)	527 (95.8)	–	–	–	–
Bloody	0 (0)	5 (0.9)	–	–	–	–
Non-watery, non-bloody	2 (1.0)	18 (3.3)	–	–	–	–
Diarrhea (times/day)			0.256	0.880	–	–
3–4	97 (49.5)	261 (47.5)	–	–	–	–
5–9	82 (41.8)	241 (43.8)	–	–	–	–
≥10	17 (8.7)	48 (8.7)	–	–	–	–
Abdominal pain			6.551	0.010	1.932	1.159–3.220
Yes	176 (89.8)	451 (82.0)	–	–	–	–
No	20 (10.2)	99 (18.0)	–	–	–	–

The novel GII.P17/GII.17 variants accounted for most of the NoV-associated AGE cases, while the GII.4 Sydney_2012 strains were previously dominant in Huzhou. We compared the clinical futures of GII.17 and GII.4 virus in the AGE patients, None significant difference were found except for the age (Additional file 1: Table S1). The proportion of children (≤5 years), young (6–15 years), adults (16–40 years) and older adults (41–60 years) and elderly patients (≥60 years) in GII.17 cases were 1.1% (1/88), 1.1% (1/88), 62.5% (55/88), 23.9% (21/88) and 11.4% (10/88), respectively. GII.4 were detected in older adults with 63.6% (7/11)). None GII.4 virus infection were founded in young (6–15 years) group, which may be due to the limited number of cases in our study.

### Genotyping and distribution of NoVs

All NoVs from samples that tested positive by real-time PCR were classified according to genotype. Regions A and C (located at RdRp and the VP1gene, respectively) were amplified with specific primers, and the PCR products were subjected to direct sequencing [22]. In total, 117 (59.69%) viruses were sequenced, and partial nucleotide sequences from two genes (capsid and RdRp) were obtained from 46 strains, which clustered into six known genotypes and one unassigned genotype: GI.P4/GI.4 (1/117, 0.85%), GI.Pb/GI.6 (1/117, 0.85%), GII.Pe/GII.4 (4/117, 4.27%), GII.P17/GII.17 (36/117, 30.77%), GII.P12/GII.3 (1/117, 0.85%), GII.P21/GII.21 (1/117, 0.85%), and GII. P unassigned /GII.13 (1/117, 0.85%; Table 2). For the remaining 71 viruses, 42 RdRp sequences and 29 VP1 sequences were obtained, respectively. Each consisted of four genotypes: GII.Pe (*n* = 4), GII.P17 (*n* = 36, 30.77%), GII.P21 (*n* = 1), and GII.P3 (*n* = 1); and GII.4 (*n* = 4), GII.17 (*n* = 16, 13.68%), GII.13 (*n* = 5), and GII.3 (*n* = 4). Overall, the new GII.P17/GII.17 variants were the dominant genotype, accounting for 75.21% of the viruses, followed by the GII.Pe/GII.4 Sydney_2012 strains (11.11%).

**Table 2:** Genotype distribution of identified norovirus strains in Huzhou from January to December 2015

Genogroup	Genotype		Number	Ratio (%)
GI	RdRp/Capsid	GI.Pb/GI.6	1	0.85%
		GI.P4/GI.4	1	0.85%
GII	RdRp/Capsid	GII.Pe/GII.4	5	4.27%
		GII.P17/GII.17	36	30.77%
		GII.P unassigned/GII.13	1	0.85%
		GII.P12/GII.3	1	0.85%
		GII.P21/GII.21	1	0.85%
	RdRp	GII.Pe	4	3.42%
		GII.P17	36	30.77%
		GII.P12	1	0.85%
		GII.P3	1	0.85%
	Capsid	GII.4	4	3.42%
		GII.17	16	13.68%
		GII.13	5	4.27%
		GII.3	4	3.42%
Total			117	100.00%

The monthly detection rates of the GII.P17/GII.17 and GII.Pe/GII.4 viruses are shown in Fig. 1. In the winter of 2014–2015, the new GII.P17/GII.17 variant first emerged in Huzhou and began to replace GII.Pe/GII.4 Sydney_2012 as the predominant strain [10]. From January to March, the GII.P17/GII.17 variant continued to predominate and kept increasing; it then declined in April and May. From October to November, the GII.Pe/GII.4 variants (Sydney 2012) re-emerged and became dominant again, indicating that the epidemiological trend in Huzhou may have changed.

### Phylogenetic analyses of NoV strains

**Fig. 2:**
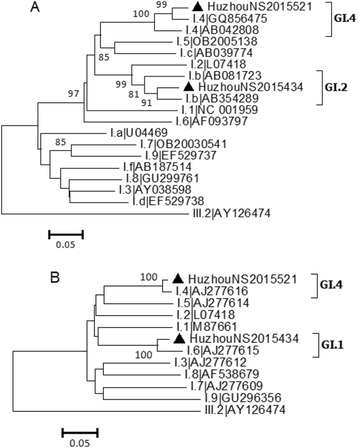
Phylogenetic analysis based on RdRp (RNA-dependent RNA polymerase) genes **a** and partial capsid protein (VP1) genes **a** of genogroup I noroviruses. Phylogenetic analysis based on RdRp genes **a** and partial VP1 genes **b** of GI NoVs. The trees were constructed by the neighbor-joining method with by the neighbor-joining method with the Maximum Composite Likelihood model in MEGA (version 6.0), validated by 1000 bootstrap replicates. Bootstrap values more than 70% was shown on the branches. NoV strains identified in Huzhou are designated by location, year and sample number (indicated by black triangles). Reference sequences are indicated by their genotypes and accession numbers. The GIII.2 strain (AY126474) was used as outgroup

**Fig. 3:**
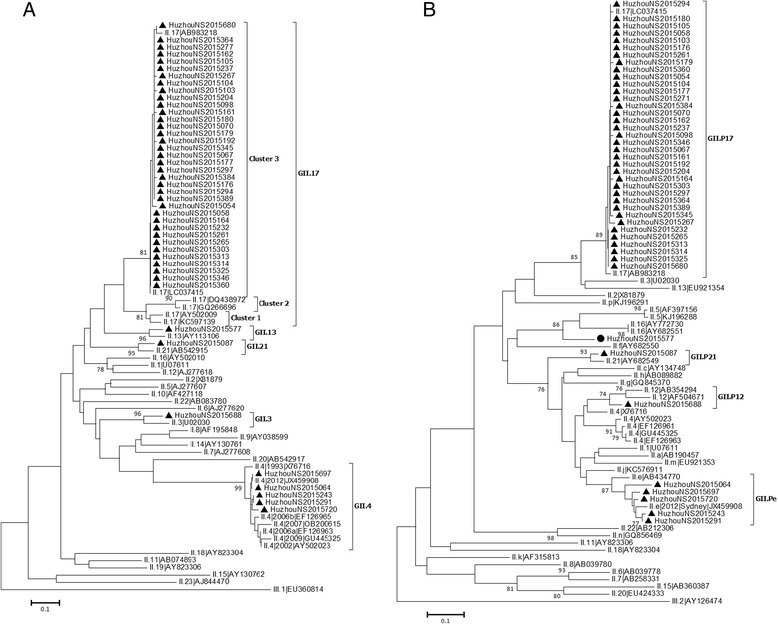
Phylogenetic analysis based on RdRp (RNA-dependent RNA polymerase) genes **a** and partial capsid protein (VP1) genes **b** of genogroup II noroviruses. Phylogenetic analysis based on RdRp genes **a** and partial VP1 genes **b** of GII NoVs. The trees were constructed by the neighbor-joining method with by the neighbor-joining method with the Maximum Composite Likelihood model in MEGA (version 6.0), validated by 1000 bootstrap replicates. Bootstrap values more than 70% was shown on the branches. NoV strains identified in Huzhou are designated by location, year and sample number (indicated by black triangles). Reference sequences (variants) are indicated by their genotypes and accession numbers. The GIII.2 strain (AY126474) was used as outgroup

In total, 46 strains with at least partial nucleotide sequences for two genes (capsid and RdRp) were subjected to a phylogenetic analysis using the MEGA software (ver. 6.06) as mentioned previously (Figs. 1 and 2). Of the two genogroup GI strains, one (HuzhouNS2015521) belonged to the GI.4 genotype, whereas the other (HuzhouNS2015434) was apparently a recombinant, for which the RdRp and capsid genes clustered with the GI.Pb and GI.6 prototype viruses, respectively. For the remaining 44 GII isolates, most of strains grouped with the novel GII.P17/GII.17 variants, which emerged as the predominant strains in Huzhou beginning in the winter of 2014. The partial sequences of the capsid gene formed a single cluster (Cluster 3 in Fig. 3b), sharing the highest identity with reference strain Kawasaki308 (accession no. LC037415). Five strains were grouped with the formerly predominant GII.4 variants GII.Pe/GII.4_Sydney_2012. The partial RdRp gene sequence of strain HuzhouNS201557 (GII.P unassigned/GII.13 in Table 2) clustered with neither GII.P16 nor GII.P5 strains and could not be assigned by the automated norovirus typing tool, indicating that a longer sequence was needed for further validation. Additionally, one GII.P21/GII.21 and one recombinant strain GII.P12/GII.3 were circulating in Huzhou in 2015.

## Discussion

In this study, we determined the levels of NoV activity in sporadic AGE from January to December 2015 in Huzhou, China. The overall prevalence of NoV infection was 26.3% (196/746). Of the 196 NoV-positive specimens, 117 were identified as NoV GII, 4 belonged to NoV GI, and 1 was a combined NoV GI and GII infection. In total, 117 viruses were sequenced, and 88 (75.21%) belonged to a novel GII.P17/GII.17 genotype. In our previous study [10], We reported the emergence and predominance of norovirus GII.17 in the same hospital from March, 2014 to February 2015. There were overlapping of using the same set of specimens of January and February 2015 between the two studies. NoV infection was detected throughout the year. The circulation peak of NoV occurred during the 2014–2015 winter–spring period.

Generally, young children (aged <5 years) and older adults (aged >60 years) are the at-risk groups for more severe NoV gastroenteritis, partly due to immature and waning immunity, respectively [23]. Our previous research found that children (≤10 years) and elderly individuals (>60 years) were more likely to be infected with GII.4 [10].

In contrast, GII.17 seemed more likely to infect people aged 16–60 years, previously considered the least vulnerable population. Chan MC [24] recently reported that the median (IQR) age of GII.17 (*n* =128) cases was significantly older than that of GII.4 (*n* =163) cases (49 (9–75) versus 1 (1–8) years, the proportion of older children and young adults (aged 5–65 years) and older adults (aged > 65 years) in GII.17 cases were higher than in GII.4 cases (47.7% vs 19.0%,36.7% vs 11.0% respectively). Adults aged 16–60 years are generally regarded as non-compromised, and thus unlikely to develop severe NoV gastroenteritis that requires medical attention. Why the adults seem more susceptible to the novel GII.P17/GII.17 virus need to be further studied.

Generally, NoV activity peaks in the winter–spring period, but we found no significant increase in the winter of 2015 compared to the summer and autumn of 2015. This may be because, after several months of exposure to NoV GII.17, the population had acquired immunity against GII.17, and the previously circulating GII.4 Sydney 2012 strain was still at low levels of activity.

Over the past two decades, NoV GII.4 variants have been responsible for the majority of both outbreaks and sporadic cases of AGE [25]. GII.4 variants have emerged every 2–3 years, and the new variants then replaced the old ones as the predominant variant. The emergence of novel GII.4 variants has caused at least six pandemics of NoV-associated acute gastroenteritis: US 95/96 (1995–1996), Farmington Hills (2002–2003), Hunter (2004–2005), Den-Haag 2006b (2006–2007), New Orleans 2009 (2009–2010), and most recently, GII.4 Sydney 2012 (2012–2013) [26–31]. The GII.4 Sydney-2012 variant (a recombination of strains GII.Pe/GII.4) was first identified in Australia in March 2012. Subsequently, various countries worldwide reported higher incidences of NoV outbreaks or illnesses during the winter of 2012–2013; most were caused by the GII.4 Sydney 2012 variant [31–33]. The success of GII.4 viruses is due to their evolution through the accumulation of mutations into drift variants that escape immunity from previous exposure [34], intra-genotype recombination of contemporary GII.4 noroviruses that foster the emergence of novel GII.4 variants [35], and alterations in binding properties [36].

In the Huzhou area, the GII.4 Sydney 2012 variant was first identified in November 2012, and became the predominant GII.4 variant soon thereafter. This variant caused several outbreaks in the Huzhou area between 2012 and 2014 [8]. Between October 2014 and June 2015, the GII.4 Sydney 2012 variant was replaced by a novel GII.P17/GII.17 variant, and the detection rate decreased greatly. In the winter of 2015, the GII.4 Sydney 2012 variant re-emerged in Huzhou. Further investigations are needed to elucidate the changing epidemiological trends of NoVs in Huzhou.

Some other genotypes, such as GII.3, GII.6, and GII.13, were also detected in Huzhou. However, none of these non-GII.4 genotypes ever replaced the GII.4 genotypes’ dominance. The novel GII.P17/GII.17 variant was first detected in October 2014 in Huzhou, and caused an increasing number of sporadic cases. During the 2014–2015 season, it became predominant, replacing the GII.4 Sydney variant from January 2015 [10]. This result is consistent with studies from other regions of China and in other countries [13–16]. This was the first time that a non-GII.4 genotype replaced the GII.4 variants as the predominant strain in Huzhou.

The GII.17 genotype has been circulating in the human population for several decades [37]. In Africa, Asia, North America, and South America, GII.17 has been detected sporadically [38–42]. According to CaliciNet, there were four reported GII.17 outbreaks between 2009 and 2013 in the United States [43]. During this period, Denmark and South Africa reported sporadic GII.17 cases on Noronet [44]. Kiulia reported that the NoV GII.17 virus accounted for 76% of all detected NoV strains in rivers in rural and urban areas in Kenya between 2012 and 2013 [45].

In the 2014–2015 season, a NoV genotype GII.P17/GII.17 variant emerged and caused outbreaks in multiple cities in Guangdong Province, China [14]. During that winter, 23 outbreaks of NoV AGE occurred; 16 were related to a new GII.17 variant in Jiangsu, China [15]. In other parts of Asia, such as Hong Kong [24] and Taiwan, the novel GII.17 also became predominant at about the same time [46]; similarly, in other countries, such as Japan and the United States, the novel GII.17 variant also caused an increase in the number of cases during the 2014–2015 season [11, 13].

However, not all NoV outbreaks or sporadic AGE cases are genotyped beyond the GI and GII classification, so the new GII.17 may have been more common than we know. Indeed, most previously available GII.17 sequences included only the 5’-end of VP1 (C region), and very few sequences covered ORF1 or ORF2 [22]. Viruses with a GII.17 VP1 genotype contain an ORF1 genotype with the GII.P13, GII.P16, GII.P3, or GII.P4 genotype [41, 47–49]. These previous GII.17 viruses did not cause an increase in NoV activity. Once the new GII.P17 RdRp gene combined with the GII.17 ORF1 gene, the new GII.P17/GII.17 then steadily replaced GII.Pe/GII.4 (GII.4 Sydney 2012) as the predominant NoV in circulation worldwide. The acquisition of the novel ORF1 may explain the sudden emergence and the widespread ability of the new GII.P17/GII.17 variant. Sequence comparisons of GII.17 variants detected in October 2014 in Huzhou showed an RNA-dependent RNA polymerase (RdRp) gene cluster with GII.P13 viruses that had not been detected before [11]. Thus, in previous studies, we assigned the new viruses as GII.P13/GII.17 [10]. This variant was ultimately assigned to the RdRp genotype, GII.P17 [13]. Thus, the strains predominant in 2014–2015 in Huzhou were in fact GII.P17/GII.17 viruses.

## Conclusions

In conclusion, we report the epidemiological patterns and genetic characteristics of NoV in Huzhou between January and December 2015, after the appearance of GII.17. We found that after the emergence of GII.17 in October 2014, it steadily replaced the previously circulating GII.4 Sydney 2012 strain, and continued to be dominant in 2015. Furthermore, our results indicate that the new GII.17 commonly infected people aged 16–60 years, who were previously considered a less vulnerable population. As NoVs are a major cause of non-bacterial gastroenteritis, systematic surveillance and evidence-based studies are important to elucidate the molecular epidemiology and spread of NoVs.

## Additional file

Additional file 1: Table S1. Epidemiological and clinical features of GII.17 and GII.4 positive acute gastroenteritis (AGE) patients, None significant difference were found except for the age after compared the epidemiological and clinical futures of GII.17 and GII.4 virus in the AGE patients. (DOC 53 kb)
