# Supplementary material for: Clinical and molecular analyses of norovirus-associated sporadic acute gastroenteritis: the emergence of GII.17 over GII.4, Huzhou, China, 2015
Source: BMC Infect Dis. 2016 Nov 29;16:717. doi: 10.1186/s12879-016-2033-x (PMC5126990; doi:10.1186/s12879-016-2033-x)
Supplement: Additional file 1: Table S1. — Epidemiological and clinical features of GII.17 and GII.4 positive acute gastroenteritis (AGE) patients, None significant difference were found except for the age after compared the epidemiological and clinical futures of GII.17 and GII.4 virus in the AGE patients. (DOC 53 kb) [file 12879_2016_2033_MOESM1_ESM.doc]

Table S1 Epidemiological and clinical features of GII.17 and GII.4 positive acute gastroenteritis (AGE) patients

| Parameter | GII.17 (N=88) | GII.4(N=11) |
| --- | --- | --- |
| Sex (male：female) | 48:41 | 5:6 |
| Age |  |  |
| ≤5 | 1 (1.1%) | 1(9.1%) |
| 6-15 | 1 (1.1%) | 0 |
| 16-40 | **55 (62.5%)** | 3(27.3%) |
| 41-60 | 21 (23.9%) | **7(63.6%)** |
| >60 | 10 (11.4%) | 0 |
| Fever (>38℃) | 2 (2.3%) | 1(9.1%) |
| Vomiting | 25 (28.4%) | 2(18.2%) |
| Watery Stool | **87 (98.9%)** | **11(100%)** |
| Diarrhea (times/day) |  |  |
| 3-4 | **45 (51.1%)** | **6(54.5%)** |
| 5-9 | 37 (42.0%) | 3(27.3%) |
| ≥10 | 6 (6.8%) | 2(18.2%) |
| Abdominal pain | **82 (93.2%)** | **8(72.7%)** |
